# Heart Failure with Preserved Ejection Fraction in Women with Breast Cancer Prior to Cancer Treatment: Insights from a Cardio-Oncology Assessment

**DOI:** 10.3390/jcm15145739

**Published:** 2026-07-22

**Authors:** Allegra Battistoni, Marco Di Francesco, Davide Bernardo, Jacopo Capparelli, Nicola Tartaglia, Simona Pisegna, Linda Piras, Gianmarco Cellammare, Pasqualino Raponi, Davide D’Anna, Raffaella Mistrulli, Damiano Magrì, Emanuele Barbato

**Affiliations:** Department of Clinical and Molecular Medicine, Sapienza University of Rome, 00185 Roma, Italyjacopo.capparelli@outlook.it (J.C.); davidedanna1997@gmail.com (D.D.); raffaella.mistrulli@uniroma1.it (R.M.);

**Keywords:** heart failure with preserved ejection fraction, breast cancer, cardiotoxicity, H_2_FPEF, ABA, HFA-PEFF, cardioncology, diastolic dysfunction, anthracyclines, anti-HER2

## Abstract

**Background:** Breast cancer patients are exposed to cardiovascular complications related to both pre-existing cardiovascular risk factors and cardiotoxic cancer therapies. Heart failure with preserved ejection fraction (HFpEF) is an emerging and often under-recognized condition in this population, potentially present before treatment initiation and further influenced by oncologic therapies. This study aimed to characterize baseline HFpEF probability and its longitudinal evolution using validated scoring systems in a real-world cardio-oncology cohort. **Methods:** We retrospectively evaluated 145 women with breast cancer undergoing baseline cardio-oncology assessment before initiation of potentially cardiotoxic therapies. HFpEF probability was estimated using three validated algorithms (H_2_FPEF, ABA, and HFA-PEFF). A subgroup of 89 patients underwent exploratory reassessment after 12 ± 3 months to investigate longitudinal changes in HFpEF probability according to treatment. **Results:** At baseline, HFpEF probability varied substantially according to the scoring system applied, with 14.8–41.5% of patients classified as having intermediate-to-high probability. Agreement among the three algorithms was limited, particularly between HFA-PEFF and H_2_FPEF/ABA scores. Exploratory longitudinal analyses showed no significant changes in HFpEF probability categories during follow-up. **Conclusions**: HFpEF probability assessment in breast cancer patients showed substantial baseline heterogeneity across validated scoring systems, with clinically relevant differences in risk classification and limited inter-score agreement. These findings provide novel insights into an underexplored cardio-oncology phenotype and suggest that currently available HFpEF scoring systems may not be directly interchangeable in this clinical setting. Prospective studies including provocative testing are needed to determine the clinical relevance of HFpEF probability scores in breast cancer patients.

## 1. Introduction

Over the last few decades, advances in cancer diagnosis and treatment have significantly improved survival among oncologic patients, leading to a growing population of long-term cancer survivors. As cancer-related mortality has decreased, cardiovascular disease (CVD) has emerged as a major determinant of morbidity and mortality in both active cancer patients and survivors [1]. Indeed, CVD and cancer share common risk factors and pathophysiological pathways, and anticancer therapies may also induce cardiac side effects [2].

Breast cancer is a key setting in cardio-oncology due to its high prevalence and the frequent use of potentially cardiotoxic therapies, including anthracyclines and anti-HER2 agents. Although these treatments have significantly improved oncologic outcomes, they are associated with an increased risk of cardiovascular toxicity, particularly heart failure with reduced ejection fraction (HFrEF) [3]. Accordingly, cardio-oncology surveillance has traditionally focused on the detection of cancer therapy-related cardiac dysfunction through serial assessment of left ventricular ejection fraction (LVEF), reflecting a predominant emphasis on systolic impairment [4].

However, growing evidence suggests that cardiovascular vulnerability in cancer patients extends beyond systolic dysfunction. Heart failure with preserved ejection fraction (HFpEF), characterized by preserved systolic function with impaired diastolic relaxation and increased filling pressures, is increasingly recognized in women with cardiometabolic comorbidities such as hypertension, obesity, diabetes mellitus, and atrial fibrillation [5]. Cancer-related inflammation, endothelial dysfunction, and oxidative stress, together with treatment-related myocardial effects, may further contribute to its development [5]. Despite its clinical relevance, HFpEF remains under-recognized in routine practice, since its diagnosis requires integration of clinical, structural, and functional parameters rather than a single marker [4,5]. To improve diagnostic accuracy, several probability-based tools have been developed [6,7], but their applicability in oncology populations remains uncertain. This represents an important gap in knowledge given the coexistence of traditional cardiovascular risk factors, cancer-related systemic effects, and treatment-induced cardiac injury in this population [1,5,8]. The primary objective of the study was to evaluate baseline HFpEF probability in women with breast cancer prior to the initiation of potentially cardiotoxic therapy using three validated scoring systems (H_2_FPEF, ABA, and HFA-PEFF) [6,7,8,9]. Secondary and exploratory objectives included the assessment of inter-score agreement, longitudinal changes in HFpEF probability at follow-up, and comparisons between treatment groups. These analyses were considered hypothesis-generating due to the observational design and limited sample size of subgroup analyses.

## 2. Materials and Methods

### 2.1. Study Population

We retrospectively enrolled consecutive female patients with histologically confirmed breast cancer referred to the cardio-oncology outpatient clinic of Sant’Andrea University Hospital for pre-treatment cardiovascular evaluation between October 2020 and March 2026. All patients underwent baseline cardio-oncology assessment according to European Society of Cardiology guidelines [4] before initiation of potentially cardiotoxic oncologic therapies, including anthracyclines and/or anti-HER2 agents, administered either alone or in combination. Inclusion criteria were: age ≥18 years, baseline cardio-oncology evaluation performed before or within 30 days from initiation of anthracycline and/or anti-HER2 therapy, and availability of clinical and echocardiographic data required for HFpEF probability score calculation. Exclusion criteria included LVEF < 50% at baseline, previously documented HFrEF, prior exposure to potentially cardiotoxic cancer therapies, and incomplete echocardiographic data precluding score assessment.

### 2.2. Study Design and Follow-Up

The baseline cohort included all eligible patients undergoing pre-treatment cardio-oncology evaluation and was used to assess baseline HFpEF probability and cardiovascular profile. A longitudinal subgroup with repeat clinical and echocardiographic assessment approximately 12 ± 3 months after treatment initiation was used for exploratory longitudinal analyses of HFpEF probability and cardiac changes over time. To explore the potential impact of different oncologic regimens on cardiac phenotype and HFpEF probability, the longitudinal cohort was further stratified according to treatment exposure into the following groups:

- Anthracycline group: patients treated with anthracycline-based chemotherapy alone (Epirubicin).
- Anti-HER2 group: patients treated with anti-HER2-based chemotherapy alone (e.g., Trastuzumab).
- Combination group (Anthracyclines + anti-HER2): patients treated with a combination of anthracyclines and anti-HER2 targeted therapies.

Comparative analyses between treatment groups were performed to explore differences in longitudinal HFpEF probability scores and echocardiographic parameters. While the primary objective of the study was the assessment of baseline HFpEF probability before initiation of cancer therapy, these longitudinal and treatment-specific analyses were considered exploratory and hypothesis-generating.

### 2.3. Clinical, Laboratory, and Electrocardiographic Assessment

Demographic, anthropometric, clinical, laboratory, electrocardiographic, and treatment data were retrospectively extracted from electronic medical records at baseline (within 30 days before initiation of oncologic therapy) in the entire cohort and at 12 ± 3 months in the longitudinal subgroup. Clinical assessment included heart failure symptoms and signs; cardiovascular risk factors and comorbidities (BMI, hypertension, diabetes mellitus, dyslipidaemia, smoking status, chronic obstructive pulmonary disease, atrial fibrillation, anaemia, coronary artery disease, and peripheral artery disease); and physical examination, blood pressure, and electrocardiographic findings. Laboratory evaluation included haemoglobin, creatinine, blood urea nitrogen, sodium, potassium, cardiac troponin I, BNP, and NT-proBNP. Estimated glomerular filtration rate (eGFR) was calculated using the 2021 CKD-EPI race-free equation.

### 2.4. Standard Echocardiography

Two-dimensional transthoracic echocardiography was performed at baseline in the entire cohort and at follow-up in the longitudinal subgroup according to current recommendations using a commercially available ultrasound system (Philips Affiniti 70, Philips Electronics, Amsterdam, The Netherland with software versions updated during the study period according to manufacturer-released upgrades starting from software release 5.0.x to 9.0.2). All examinations followed a standardized acquisition protocol, and echocardiographic data were retrospectively retrieved from electronic medical records. Left ventricular dimensions, wall thickness, and mass were measured according to current recommendations. LVEF was calculated using the biplane modified Simpson method. Diastolic function was assessed according to European Association of Cardiovascular Imaging recommendations [10], including left atrial volume index (LAVI), transmitral inflow (E/A), tissue Doppler septal and lateral e′ velocities, mean E/e′ ratio, and systolic pulmonary artery pressure (sPAP).

### 2.5. Heart Failure with Preserved Ejection Fraction Probability Assessment

Given the diagnostic complexity of HFpEF and the heterogeneity of currently available diagnostic frameworks, HFpEF probability was retrospectively assessed at baseline and follow-up using three validated scoring systems (H_2_FPEF, HFA-PEFF, and ABA). This multi-score approach was adopted to provide a comprehensive characterization of HFpEF probability and to evaluate the degree of concordance among different diagnostic models. As recognized by current heart failure guidelines, individual scoring systems integrate different clinical, echocardiographic, and biomarker domains and may therefore lead to different risk classifications in the same patient [11].

H_2_FPEF Score: This clinical and dynamic-based score incorporates BMI, number of hypertension medicines, atrial fibrillation, pulmonary hypertension, age and filling pressures. Patients were categorized as having a low (0–1 points), intermediate (2–5 points), or high (6 points) probability of HFpEF [12]. In addition, the score was also analyzed as a continuous variable to account for its graded relationship with disease probability. Patients were categorized as having a low (0–50%), intermediate (50–75%), or high (75%) probability of HFpEF [6].

ABA Score: This is a simplified clinical model based on BMI, age, and history of atrial fibrillation, used to estimate HFpEF probability. Patients were classified as low (<50%), intermediate (50–75%), or high (>75%) probability [12].

HFA-PEFF Score: This diagnostic algorithm, developed by the Heart Failure Association of the European Society of Cardiology, integrates functional, morphological, and biomarker domains to estimate HFpEF probability. Patients were classified as having a low (0–1), intermediate (2–4), or high (≥5) probability of HFpF [12]. The biomarker domain was calculated using BNP or NT-proBNP values according to guideline-recommended criteria when available [12]. When natriuretic peptide levels were available, the full score was calculated according to guideline-recommended criteria. In cases of missing biomarker data, patients were retained in the analysis and scored using the available functional and morphological domains to allow a pragmatic real-world comparison across scoring systems. Specific sub-analyses were conducted within the patient subgroups with and without available biomarkers to evaluate potential differences in risk classification and inter-score agreement.

### 2.6. Statistical Analysis

#### 2.6.1. Descriptive Statistics

Data distribution was assessed using the Shapiro-Wilk test and visual inspection of Q-Q plots. Baseline characteristics are summarized using appropriate descriptive statistics. Continuous variables are expressed as mean ± standard deviation or median with interquartile range, as appropriate based on distribution. Categorical variables, including cardiovascular risk factors, comorbidities, and pharmacological treatments, are reported as absolute numbers and percentages.

#### 2.6.2. Comparison and Correlation of HFpEF Probability Scores at Baseline

The relationship among the three diagnostic algorithms (H_2_FPEF, ABA, and HFA-PEFF) in the total baseline cohort was assessed using Spearman’s rank correlation coefficient. Agreement in HFpEF probability classification (low, intermediate, and high) was evaluated using weighted Cohen’s kappa coefficient, accounting for the ordinal nature of the categories. To investigate the impact of natriuretic peptide availability on HFA-PEFF classification, baseline clinical characteristics and echocardiographic parameters were compared between patients with and without available biomarker data using the Wilcoxon rank-sum test for continuous variables and the Chi-squared (χ^2^) test or Fisher’s exact test for categorical variables, as appropriate. The effect of biomarker availability on HFA-PEFF risk distribution was assessed using the Fisher–Freeman-Halton exact test. Finally, concordance among HFA-PEFF, point- and percentage-based H_2_FPEF, and ABA scores was evaluated separately according to biomarker availability using weighted Cohen’s kappa coefficients. Agreement was interpreted according to the Landis and Koch criteria, with exact two-sided *p*-values calculated and *p* < 0.05 considered statistically significant.

#### 2.6.3. Baseline Comparison of the Longitudinal Subgroup

To assess baseline comparability across treatment groups in the longitudinal cohort, continuous variables with a normal distribution were compared using one-way ANOVA, with variance homogeneity assessed by Bartlett’s test. Post hoc pairwise comparisons were performed with Bonferroni correction when appropriate. Non-normally distributed variables or variables with limited sample size (e.g., cardiac biomarkers) were compared using the Kruskal–Wallis test. Categorical variables were analyzed using Fisher’s exact test due to the small anti-HER2 subgroup (*n* = 8) and low expected frequencies in contingency tables. Given the exploratory nature of these subgroup analyses and the limited sample size, no adjustment for multiple comparisons was applied.

#### 2.6.4. Longitudinal Exploratory Analyses

Longitudinal analyses were considered exploratory and hypothesis-generating, as the primary objective of the study was the assessment of baseline HFpEF probability. Changes in clinical, echocardiographic, and laboratory parameters between baseline and follow-up were evaluated within treatment groups using paired Student’s *t*-test or Wilcoxon signed-rank test, as appropriate.

Longitudinal changes in categorical and ordinal HFpEF probability classifications were assessed using the Stuart–Maxwell test and ordinal logistic regression models, including treatment group and baseline score as covariates.

For the H_2_FPEF percentage-based score, temporal trajectories were further evaluated using mixed-effects models adjusted for baseline values. Given the exploratory nature of these analyses, the limited sample size of the follow-up cohort, and the absence of correction for multiple comparisons, longitudinal findings should be interpreted as hypothesis-generating.

Statistical analyses were performed using Stata^®^ version 18.0 (StataCorp LLC, College Station, TX, USA), with *p* < 0.05 considered statistically significant.

## 3. Results

### 3.1. Total Baseline Cohort

#### 3.1.1. Distribution of Baseline Heart Failure with Preserved Ejection Fraction Probability Scores

**Figure 1:**
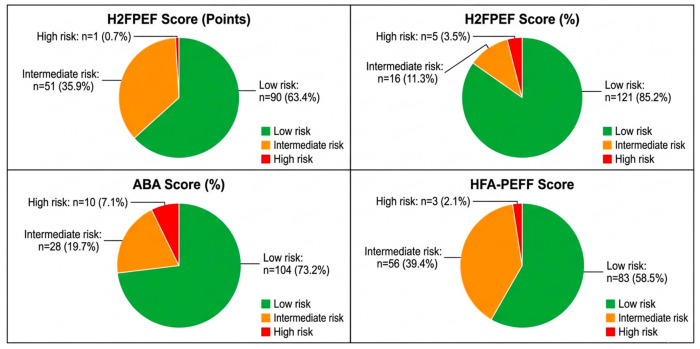
Baseline risk stratification according to heart failure with preserved ejection fraction probability scores. Biomarker data were available for 44% of the patients of the total baseline cohort.

A total of 145 female patients were included in the total baseline cohort. The mean age of the study population was 57.1 ± 12.1 years. Regarding anthropometric characteristics, the cohort presented a mean BMI of 25.4 ± 4.9 kg/m^2^. After excluding three patients due to missing BMI data (*n* = 142), the distribution of patients across low-, intermediate-, and high-probability categories varied according to the scoring system used (Figure 1). For the low-probability category, the probability-based H_2_FPEF score classified the largest proportion of patients (85.2%, *n* = 121), followed by the ABA score (73.2%, *n* = 104) and the points-based H_2_FPEF score (63.4%, *n* = 90). The HFA-PEFF algorithm assigned the lowest proportion of patients to this category (58.5%, *n* = 83). A different pattern was observed for the intermediate-probability category, which was most frequently identified by the HFA-PEFF score (39.4%, *n* = 56), followed by the points-based H_2_FPEF score (35.9%, *n* = 51). Lower proportions were observed with the ABA score (19.7%, *n* = 28) and the probability-based H_2_FPEF score (11.3%, *n* = 16). For the high-probability category, the ABA score classified 7.1% of patients (*n* = 10), followed by the probability-based H_2_FPEF score (3.5%, *n* = 5) and the HFA-PEFF algorithm (2.1%, *n* = 3), while the lowest proportion was observed using the points-based H_2_FPEF score (0.7%, *n* = 1). Of note, complete biomarker data required for calculation of the HFA-PEFF score were available in 44.0% of the baseline population. The availability of natriuretic peptides significantly influenced HFA-PEFF risk classification, resulting in a shift from lower to higher probability categories when biomarker data were available (Table S1). Overall, agreement was strong between H_2_FPEF and ABA scores, whereas concordance with HFA-PEFF remained limited (κ 0.152–0.252; detailed in Table S2).

#### 3.1.2. Cardiovascular Risk Factors, Comorbidities and Cardiovascular Therapies

The study population showed a substantial burden of baseline cardiovascular risk factors and comorbidities (Table 1 and Table S3). Obesity was present in 20.4% of patients, arterial hypertension in 33.1%, dyslipidaemia in 24.1%, diabetes mellitus in 9.0%, and a history of atrial fibrillation in 3.4%. Importantly, a considerable proportion of patients with hypertension (43.6%) and dyslipidaemia (51.4%) were not receiving guideline-directed medical therapy (GDMT) at the pre-treatment cardio-oncology evaluation. Renal function was generally preserved (mean eGFR 91.3 ± 15.0 mL/min/1.73 m^2^), while anaemia was present in 40.2% of patients with available data. Most patients were asymptomatic (93.1% NYHA class I), and baseline use of cardioprotective therapies was limited, with angiotensin-converting enzyme inhibitors or angiotensin receptor blockers (ACEi/ARB), beta-blockers, and statins prescribed in 22.8%, 16.6%, and 17.2% of patients, respectively. Natriuretic peptides were available only in a subset of the cohort, with median NT-proBNP and BNP values of 116.0 pg/mL (IQR 48.0–325.0) and 24.7 pg/mL (IQR 12.0–54.0), respectively.

#### 3.1.3. Baseline Echocardiographic Assessment

Baseline echocardiographic assessment demonstrated preserved left ventricular systolic function across the cohort, with a mean LVEF of 64.0 ± 4.8%. Further evaluation of structural and functional parameters relevant to HFpEF revealed a mean Left Ventricular Mass Index (LVMI) of 67.5 ± 16.5 g/m^2^. Only 1.4% of patients (*n* = 2) met the major criterion for left ventricular hypertrophy (LVMI ≥ 122 g/m^2^), while an additional 3.5% *(n* = 5) fell within the intermediate range (95–122 g/m^2^). The mean relative wall thickness (RWT) was 0.4 ± 0.1, and 41 patients (28.3%) exceeded the 0.42 threshold suggestive of concentric remodeling. Mean interventricular septal (IVS) thickness was 9.3 ± 1.5 mm, with 7.6% of patients (*n* = 11) showing a wall thickness ≥12 mm. Assessment of diastolic function and filling pressures-core components of both the HFA-PEFF and H_2_FPEF scoring systems showed a mean LAVI of 23.7 ± 7.2 mL/m^2^. Although the overall average remained within normal limits, 6.7% of patients (*n* = 7) had an LAVI ≥ 34 mL/m^2^, fulfilling a major diagnostic criterion, while 12.4% (*n* = 18) were classified within the intermediate range (29–33 mL/m^2^). The mean E/e′ ratio was 7.8 ± 2.1; only one patient (0.7%) exhibited a value ≥15, indicative of elevated filling pressures, whereas 30.3% (*n* = 44) presented intermediate values between 9 and 14. Reduced annular velocities (septal e′ < 7 cm/s and/or lateral e′ < 10 cm/s), consistent with impaired myocardial relaxation, were identified in 51 patients (35.2%). Finally, the mean sPAP was 26.8 ± 3.4 mmHg, with 2.1% of the cohort (*n* = 3) exceeding the 35 mmHg threshold, a relevant parameter within the H_2_FPEF score (Table 2).

### 3.2. Longitudinal Subgroup

#### 3.2.1. Cardiovascular Risk Factors, Comorbidities and Cardiovascular Therapies

Baseline HFpEF probability assessment represents the primary analysis of the present study, whereas longitudinal and treatment-related analyses are reported as exploratory. The longitudinal analysis included 89 patients with available clinical and echocardiographic reassessment at 12 ± 3 months after baseline. Patients were stratified according to treatment exposure into anthracycline monotherapy (*n* = 46), combination therapy with anthracyclines and anti-HER2 agents (*n* = 35), and anti-HER2 monotherapy (*n* = 8) (Table S4). No permanent treatment discontinuations occurred during follow-up. Baseline and follow-up characteristics are reported in Tables S5 and S6. Baseline clinical characteristics, cardiovascular medications, comorbidities, renal function, cardiac biomarkers, and echocardiographic parameters, including diastolic function indices, were comparable among treatment groups (all *p* > 0.05).

At follow-up, a notable shift in cardiovascular therapies was observed over time. In the Anthracycline-only cohort, there was a statistically significant increase in the use of beta-blockers (from 13.0% at baseline to 32.6% at follow-up; *p* = 0.029) and statin therapy (from 13.0% to 23.9%; *p* = 0.025). A significant increase in statin prescription was also observed in the Combination group (from 8.6% to 22.9%; *p* = 0.025). In contrast, within the Anti-HER2 cohort, no statistically significant longitudinal changes in cardiovascular medical therapy were observed. Finally, plasma concentrations of cardiac biomarkers (NT-proBNP and BNP) and renal function, assessed via eGFR, remained stable without significant variations over the follow-up (all *p* > 0.05). Of note, the use of endocrine therapy and radiotherapy was comparable across treatment groups, with no statistically significant differences (endocrine therapy: *p* = 0.425; radiotherapy: *p* = 0.295).

#### 3.2.2. Echocardiographic Assessment

No significant longitudinal changes in cardiac structure or function were observed at follow-up in any of the three treatment groups. Left ventricular systolic function remained preserved throughout the study period, with a stable LVEF in the Anthracycline (*p* = 0.595), Anti-HER2 (*p* = 0.688), and Combination groups (*p* = 0.959). Parameters reflecting left ventricular remodeling also remained stable. IVS thickness and RWT showed no significant longitudinal variations (all *p* > 0.05). Indices of diastolic function and filling pressures also demonstrated substantial stability over time. LAVI remained unchanged in the Anthracycline (*p* = 0.940), Anti-HER2 (*p* = 0.807), and Combination groups (*p* = 0.783). Similarly, the mean E/e′ ratio, sPAP and prevalence of impaired tissue Doppler velocities showed no significant variation (all *p* > 0.05).

#### 3.2.3. HFpEF Probability Scores in the Longitudinal Subgroup Stratified by Treatment Exposure

Longitudinal tracking of HFpEF probability showed no overall significant changes in HFpEF probability categories across all scoring systems between baseline and follow-up (Table S7). Risk scores revealed distinct trajectories across the three treatment cohorts from baseline to follow-up. Results are summarized in Table 3.

(a) H_2_FPEF Score

Anthracycline & Combination Groups: Both point- and percentage-based H_2_FPEF scores demonstrated high temporal stability. The vast majority of patients remained in the low-risk category at follow-up, with only marginal shifts toward intermediate risk.

Anti-HER2 Group: While the point-based score was entirely stable over time, the percentage-based score revealed a reduction in low-risk patients (75.0% to 62.5%) and a proportional increase in intermediate HFPEF risk probability (25.0% to 37.5%).

(b) ABA Score

Anthracycline & Combination Groups: Both cohorts exhibited a slight longitudinal shift toward higher risk. This was characterized by a minor reduction in the low-risk populations alongside modest increases in the high-risk categories (from 8.7% to 10.9% in the Anthracycline group, and 5.7% to 8.6% in the Combination group).

Anti-HER2 group: Risk distribution remained completely unaltered between baseline and follow-up (50.0% low, 37.5% intermediate, 12.5% high).

(c) HFA-PEFF Score

Anti-HER2 Group: Significant structural shifts were observed. The low-risk population declined (37.5% to 25.0%), driving a surge in intermediate-risk classification (50.0% to 75.0%). Notably, baseline high-risk HFPEF patients (12.5%) were reclassified to lower tiers at follow-up (0.0%).

Anthracycline Group: This group demonstrated a mild progression toward intermediate risk (37.0% to 41.3%), with a corresponding decrease in low-risk patients and a stable high-risk prevalence (2.2%).

Combination Group: This group exhibited a divergent trend compared to the other cohorts, showing an overall expansion of the low-risk tier (54.3% to 60.0%) and a reduction in intermediate-risk cases, despite a marginal increase in the high-risk stratum (2.9% to 5.7%).

Of note, these findings describe aggregate shifts in risk stratification within each treatment cohort and do not depict individual patient-level trajectories.

In the subgroup with available HFA-ICOS classification and longitudinal follow-up (*n* = 41), baseline HFA-ICOS risk categories were not significantly associated with longitudinal changes in HFpEF probability as assessed by the H_2_FPEF, HFA-PEFF, or ABA scores (all *p* > 0.05). However, these analyses should be interpreted cautiously because most patients were classified as low HFA-ICOS risk, resulting in limited statistical power.

#### 3.2.4. Longitudinal Subgroup: H_2_FPEF Risk Score Analysis

As an exploratory analysis, longitudinal changes in H_2_FPEF (points-based) risk categories were evaluated using ordered logistic regression. The analysis was restricted to the Anthracycline monotherapy (*n* = 46) and Combination therapy groups (*n* = 35), given the limited sample size of the anti-HER2 monotherapy group (*n* = 8). Baseline characteristics were comparable between the two groups (Table S5). In the multivariable model, the follow-up H_2_FPEF score category was treated as the ordinal dependent variable, while treatment group (Combination therapy vs. Anthracycline monotherapy) and baseline H_2_FPEF score were included as independent predictors. The model showed a strong and highly significant association between baseline and follow-up risk classification, with each unit increase in baseline H_2_FPEF score associated with substantially higher odds of a worse risk category at follow-up (odds ratio [OR]: 21.01; 95% confidence interval [CI]: 8.81–50.10; *p* < 0.001). Combination therapy was independently associated with higher follow-up H_2_FPEF point-based risk categories compared with anthracycline monotherapy (OR: 3.00; 95% CI: 1.09–8.26; *p* = 0.033). The model showed adequate performance and fulfilled the proportional odds assumption. However, longitudinal changes in H_2_FPEF probability scores (percentage-based) did not differ between treatment groups (*p* = 0.673). Consistently, mixed-effects and ANCOVA models showed no significant effect of time, treatment group, or their interaction, indicating stable and parallel trajectories during follow-up.

## 4. Discussion

Breast cancer survival has improved substantially, making CVD a major determinant of long-term outcomes among survivors [1,13]. Traditional cardiovascular risk factors frequently coexist with cancer, increasing susceptibility to treatment-related cardiac injury [14]. Breast cancer is a paradigmatic cardio-oncology setting due to the widespread use of potentially cardiotoxic therapies, particularly anthracyclines and anti-HER2 agents [8,15]. Cardio-oncology surveillance has traditionally focused mainly on LVEF for early detection of systolic dysfunction [1,4,8], but this approach may underestimate HFpEF, a clinically relevant syndrome, particularly in women with cardiometabolic comorbidities [5]. Indeed, available evidence suggests a baseline HFpEF prevalence of 5.7% among women with breast cancer, while the incidence after cancer treatment ranges from 6.7% to 14.6% [5,16]. Cancer-related inflammation, endothelial dysfunction, and oxidative stress, together with treatment-induced myocardial injury, may promote HFpEF development or progression [1,8,10,17,18,19,20]. Accordingly, recent statements from the Heart Failure Society of America [1] and the Heart Failure Association of the European Society of Cardiology [4] recommend that cardiovascular assessment in oncology should include evaluation of diastolic function, filling pressures, left atrial remodeling, pulmonary pressures, and natriuretic peptides [1,4,5]. However, HFpEF phenotyping remains poorly explored in cardio-oncology populations, and the applicability of currently available diagnostic algorithms in this setting is unknown. Within this framework, our study provides a real-world assessment of baseline HFpEF probability and cardiovascular risk profile in women with breast cancer undergoing pre-treatment cardio-oncology evaluation.

### 4.1. Baseline HFpEF Probability Assessment: Divergence Between Diagnostic Scoring Systems and Clinical Implications

At baseline, HFpEF probability classification varied substantially according to the scoring system applied, with HFA-PEFF identifying a higher proportion of intermediate-risk patients compared with H_2_FPEF and ABA. Overall, H_2_FPEF and ABA showed stronger concordance, whereas agreement with HFA-PEFF was limited (κ 0.152–0.252) (Figure 1).

These findings are consistent with the known conceptual and methodological differences among the three diagnostic frameworks. HFpEF is increasingly recognized as a heterogeneous syndrome encompassing multiple phenotypes and pathophysiological pathways, with diagnostic scores weighting clinical, structural, functional, and biomarker domains differently [7,21]. In this context, the H_2_FPEF and ABA scores-both mainly driven by clinical variables such as age, BMI, and atrial fibrillation-showed more aligned and stable classifications, as reflected by their strong correlation in our cohort. In contrast, the HFA-PEFF algorithm applies a broader multiparametric approach, including echocardiographic structural criteria and natriuretic peptides, which tends to shift a larger proportion of patients toward the intermediate-probability category, particularly in cases with incomplete or borderline phenotypes. The availability of natriuretic peptides represents an important methodological issue for the application of the HFA-PEFF algorithm in real-world cardio-oncology practice. Although missing biomarkers influenced risk redistribution, sensitivity analyses in patients with complete biomarker assessment confirmed that the limited concordance with H_2_FPEF and ABA scores was not solely attributable to incomplete data, but also reflected intrinsic differences in score architecture and weighting of clinical, echocardiographic, and biomarker domains (Tables S1 and S2). These findings highlight the challenges of applying HFpEF probability tools in oncology populations, where treatment-related factors and baseline cardiometabolic vulnerability may influence phenotypic characterization. Therefore, while natriuretic peptides may improve risk refinement, no single score should be considered sufficient for HFpEF phenotyping in cardio-oncology, supporting a multiparametric approach integrating clinical, imaging, and biomarker information.

Beyond the previously discussed limitations of the HFA-PEFF score, other risk scores also exhibit specific limitations in routine cardio-oncology assessment, such as the ABA score, that may oversimplify risk stratification due to its reliance on a restricted set of clinical variables. Conversely, the H_2_FPEF score appears to provide a more balanced integration of clinical and echocardiographic parameters, offering greater robustness in real-world oncologic populations where complete datasets are often unavailable.

Although the prognostic significance of HFpEF probability scores in cancer populations remains to be established, evidence from non-oncologic cohorts indicates that both H_2_FPEF and HFA-PEFF scores are associated with incident HFpEF, cardiovascular hospitalization, and mortality [6,22]. The clinical relevance of HFpEF in breast cancer is further supported by emerging epidemiological data. In a large population-based analysis, Kwan et al. [23] demonstrated that women with breast cancer had a significantly higher risk of developing HFpEF compared with matched controls, with a less pronounced increase in HFrEF risk. These findings suggest that HFpEF may represent an under-recognized cardiovascular phenotype among breast cancer survivors. However, routine cardio-oncology surveillance remains largely centered on LVEF, potentially overlooking early abnormalities in diastolic function or filling pressures. Early phenotypic identification may therefore represent an opportunity to optimize preventive strategies [24]. This is particularly relevant in oncology patients, in whom HFpEF-related symptoms such as fatigue and exertional dyspnea are difficult to interpret due to the frequent coexistence of anemia, systemic inflammation, and deconditioning [25]. Accordingly, the HFpEF probability distribution observed in our chemo-naïve cohort likely reflects the combined contribution of baseline cardiovascular vulnerability and the limitations of currently available diagnostic frameworks when applied to a complex oncologic population.

### 4.2. Pre-Treatment Cardiovascular Risk Burden

The cardiovascular profile of our cohort confirms that women with breast cancer frequently present with traditional risk factors, including hypertension, obesity, dyslipidaemia, and diabetes mellitus, with prevalences comparable to contemporary cohorts and consistent with shared cardiometabolic determinants between cancer and CVD [4,26]. Notably, a substantial proportion of patients lacked optimal preventive therapy, with nearly half of hypertensive and more than half of dyslipidaemic patients not receiving GDMT at baseline, in line with reported gaps in cardiovascular prevention even in cardio-oncology settings [27,28,29]. In addition, natriuretic peptides were available in less than half of patients, limiting the applicability of biomarker-dependent algorithms such as HFA-PEFF and reflecting the suboptimal implementation of guideline-recommended biomarker assessment [1,4,30]. Overall, these findings highlight the need for a more comprehensive baseline cardiovascular evaluation, including systematic biomarker assessment, to better identify patients with possible subclinical HFpEF.

### 4.3. Longitudinal Clinical, Echocardiographic, and Pharmacological Trajectories

The longitudinal analysis, primarily involving Anthracycline (*n* = 46) and Combination Therapy (*n* = 35) groups, showed comparable baseline characteristics. During follow-up, LVEF and major echocardiographic parameters remained stable across groups, indicating no relevant early changes in systolic or diastolic phenotype. Exploratory analyses showed increased use of cardioprotective medications (BB, ACEi/ARB, and statins), particularly in the Anthracycline group, possibly reflecting greater cardio-oncologic attention during treatment. However, this pharmacological intensification remained only partially aligned with the baseline cardiovascular risk burden, characterized by a high prevalence of hypertension (33.1%), dyslipidaemia (24.1%), and obesity (20.4%), with frequent undertreatment of modifiable risk factors, consistent with previous reports [31]. Biomarkers and renal function remained stable during follow-up. The absence of early changes should be interpreted considering that cardiovascular effects related to endocrine therapy and radiotherapy may emerge over longer timeframes [4,32]; these exposures were similarly distributed between treatment groups in our cohort. Overall, these findings are exploratory and highlight persistent gaps in the implementation of guideline-directed cardiovascular prevention in breast cancer patients.

### 4.4. Treatment Effect and Longitudinal Stability of HFpEF Risk Scores

Overall, longitudinal analysis showed no significant changes in HFpEF probability categories across all scoring systems between baseline and follow-up, indicating short-term stability of risk classification (Table S7). These findings are exploratory and hypothesis-generating. Although multivariable analysis identified an association between Combination Therapy and higher follow-up H_2_FPEF point-based risk compared with Anthracycline monotherapy (OR 3.00; 95% CI 1.09–8.26; *p* = 0.033), this isolated finding should be interpreted with caution in light of the overall neutral longitudinal results. However, intra-group trajectories were overall minimal, particularly for the H_2_FPEF score, which showed near-identical low-risk proportions over time in both the Anthracycline (89.1% vs. 89.1%) and Combination groups (88.6% vs. 88.6%). The points-based H_2_FPEF score showed only marginal shifts toward intermediate risk, while high-risk categories remained negligible. Overall, the H_2_FPEF score showed the most stable longitudinal behavior, although this observation should be interpreted cautiously given the exploratory nature of the analyses. In contrast, HFA-PEFF and ABA scores showed greater variability, reflecting intrinsic methodological limitations. The greater variability observed with the HFA-PEFF score was likely influenced by missing natriuretic peptides (~55%), a known limitation in real-world cardio-oncology practice despite guideline recommendations [7]. Similarly, the ABA score showed a seemingly paradoxical increase in high-risk classification over time in both groups (Anthracycline: 8.7% to 10.9%; Combination: 5.7% to 8.6%). However, this pattern is most likely not reflective of true cardiovascular deterioration but rather of a methodological artifact inherent to the score’s structure. Given that the ABA score relies exclusively on age, BMI, and atrial fibrillation history, longitudinal changes are essentially driven by aging and small fluctuations in body weight. In oncology populations, however, BMI is particularly vulnerable to non-cardiac influences. Corticosteroid-based supportive therapy, chemotherapy-related nausea, anorexia, and cancer-associated cachexia may all induce clinically relevant weight fluctuations, thereby influencing longitudinal interpretation of BMI-based risk tools. These mechanisms limit the specificity of BMI-driven risk scores in oncologic cohorts and may generate apparent risk transitions that do not correspond to true changes in cardiac phenotype. From a pathophysiological perspective, the absence of short-term changes is not unexpected. Anthracycline and anti-HER2 therapies induce early subclinical myocardial injury preceding structural remodeling [3], while radiotherapy- and endocrine-related cardiovascular effects typically evolve over longer timeframes [32,33,34]. HFpEF itself is a chronic syndrome driven by cumulative inflammation, endothelial dysfunction, and ventricular–vascular uncoupling [7,18]. Accordingly, the lack of early variation likely reflects temporal latency rather than true stability. Consistently, epidemiological data show delayed but sustained heart failure risk in breast cancer survivors [28,35]. Given the exploratory nature of these analyses, the limited sample size, and the relatively short follow-up, these findings should be interpreted as describing short-term phenotypic stability rather than evidence of treatment-related differences or long-term cardiovascular safety.

### 4.5. Limitations

Several limitations should be acknowledged. First, this was a single-center retrospective study, and therefore the findings may not be fully generalizable to broader breast cancer populations. Second, the sample size was limited, particularly for patients receiving anti-HER2 therapy alone, reducing the power of subgroup analyses. Third, natriuretic peptide measurements were unavailable in a proportion of patients, potentially limiting the applicability of the HFA-PEFF algorithm; however, sensitivity analyses in patients with complete biomarker data confirmed the consistency of the main findings. Fourth, in the longitudinal cohort, although most treatment characteristics were available, cumulative anthracycline exposure, detailed anti-HER2 treatment duration, and temporary non-cardiac treatment interruptions were not systematically collected; therefore, treatment-related longitudinal analyses should be considered exploratory. Fifth, the relatively short follow-up may have limited the detection of late cardiovascular effects related to radiotherapy, endocrine therapy, and chronic cardiometabolic remodeling. Finally, confirmatory HFpEF assessments, including diastolic stress echocardiography or invasive hemodynamic evaluation, were not systematically available; thus, HFpEF probability was based on validated non-invasive scores rather than definitive confirmation. Accordingly, given the observational design, causal relationships between anticancer treatments and longitudinal changes in HFpEF probability cannot be established.

## 5. Conclusions

The principal finding of this study is that HFpEF probability assessment in women with breast cancer is highly dependent on the diagnostic algorithm applied, leading to substantial variability in baseline risk stratification. Although a relevant proportion of patients showed an intermediate or high probability of HFpEF before initiation of potentially cardiotoxic therapies, the three validated scoring systems frequently classified the same population differently. At the same time, this cohort exhibited a considerable burden of cardiovascular risk factors, often without optimal preventive treatment, reinforcing the need for a more comprehensive baseline cardiovascular evaluation. By contrast, exploratory longitudinal analyses showed no major changes in HFpEF probability during the first year of follow-up, suggesting early phenotypic stability rather than long-term cardiovascular safety. To our knowledge, this is one of the first studies specifically evaluating and comparing HFpEF probability scoring systems in a pre-treatment breast cancer cardio-oncology population. This addresses an important gap in current evidence, as these diagnostic algorithms were developed and validated outside the cardio-oncology setting, where the coexistence of cancer-related inflammation, cardiometabolic comorbidities, and planned cardiotoxic therapies may influence both HFpEF probability estimation and agreement between scores. A comprehensive application of HFpEF probability scores, including systematic assessment of the required clinical, echocardiographic, and biomarker domains, may improve baseline cardiovascular phenotyping and help identify patients who may require further diagnostic evaluation, including provocative testing. This approach may support optimization of cardiovascular risk factors and a more tailored clinical assessment in selected patients. Future prospective studies with larger sample sizes, systematic biomarker collection, confirmatory diagnostic approaches, and longer follow-up are needed to determine whether baseline HFpEF probability scores are associated with clinically meaningful outcomes.

## Figures and Tables

**Table 1:** Total baseline cohort characteristics.

Variable	Patients (*n* = 145)
Mean Age, years ± SD	57.1 ± 12.1
Mean body mass index (BMI), kg/m^2^ ± SD	25.4 ± 4.9
BMI * ≥ 30, *n* (%)	29 (20.4)
Smoke habit, *n* (%)	43 (29.7)
Anaemia *, *n* (%)	45 (40.2)
Atrial fibrillation history, *n* (%)	5 (3.4)
**Coexisting illness**	
Hypertension, *n* (%)	48 (33.1)
Patients not on GDMT, *n* (%)	21 (43.6)
Dyslipidemia, *n* (%)	35 (24.1)
Patients not on GDMT, *n* (%)	18 (51.4)
Diabetes, *n* (%)	13 (9.0)
Patients not on GDMT, *n* (%)	3 (23.1)
COPD, *n* (%)	1 (0.7)
**Renal function**	
eGFR *, mL/min/1.73 m^2^ SD	91.3 ± 15.0
eGFR ≤ 60 mL/min/1.73 m^2^, *n* (%)	3 (2.9)
**NYHA Class, *n* (%)**	
I, *n* (%)	135 (93.1)
II, *n* (%)	9 (6.2)
III, *n* (%)	1 (0.7)
**HFA-ICOS Risk Assessment score**	
Low risk, *n* (%)	26 (63.4)
Medium risk, *n* (%)	13 (31.7)
High risk, *n* (%)	2 (4.9)
Very high risk, *n* (%)	0
**Ongoing pharmacological treatment**	
ACEi/ARB, *n* (%)	33 (22.8)
BB, *n* (%)	24 (16.6)
Calcium antagonists, *n* (%)	0 (0)
MRA, *n* (%)	0 (0)
SGLT2-i, *n* (%)	1 (0.7)
Statin therapy, *n* (%)	25 (17.2)
**Biomarkers**	
Median NT-proBNP, pg/mL (IQR)	116.0 (48.0–325.0)
Median BNP, pg/mL (IQR)	24.7 (12.0–54.0)

* For BMI, anemia and eGFR ≤ 60 mL/min/1.73 m^2^, absolute frequencies and percentages were calculated based on the total number of patients with available data for each specific variable.

**Table 2:** Total baseline cohort echocardiographic assessment.

Variable	Patients (*n* = 145)
LVEF, % ± SD	64.0 ± 4.8
LVEDD mm ± SD	44.6 ± 4.3
IVS, mm ± SD	9.3± 1.5
IVS ≥ 12 mm, *n* (%)	11 (7.6)
RWT, *n* ± SD	0.4 ± 0.1
RWT ≥ 0.42, *n* (%)	41 (28.3)
LAVI, mL/m^2^ ± SD	23.7 ± 7.2
LAVI ≥ 34 mL/m^2^, *n* (%)	7 (6.7)
LAVI 29–33 mL/m^2^, *n* (%)	18 (12.4)
E/e’, *n* ± SD	7.8 ± 2.1
E/e’ ≥ 15, *n* (%)	1 (0.7)
E/e’ 9–14, *n* (%)	44 (30.3)
Septal e’ < 7 cm/s or lateral e’ < 10 cm/s, *n* (%)	51 (35.2)
sPAP, mmHg ± SD	26.8 ± 3.4
s ≥ 35 mmHg, *n* (%)	3 (2.1)
LVMI, g/m^2^ ± SD	67.5 ± 16.5
LVMI ≥ 122 g/m^2^, *n* (%)	2 (1.4)
LVMI 95–122 g/m^2^, *n* (%)	5 (3.5)

**Table 3:** HFpEF Probability Scores in the Longitudinal Subgroup Across Treatment Groups.

	Anthracycline Group (*n* = 46)		Anti-HER2 Group (*n* = 8)		Combination Group (*n* = 35)	
H_2_FPEF Score (points)						
	Baseline	Follow-up	Baseline	Follow-up	Baseline	Follow-up
Low risk, *n* (%)	32 (69.6)	31 (67.4)	5 (62.5)	5 (62.5)	22 (62.9)	21 (60.0)
Intermediate risk, *n* (%)	13 (28.3)	14 (30.4)	3 (37.5)	3 (37.5)	13 (37.1)	14 (40.0)
High risk, *n* (%)	1 (2.2)	1 (2.2)	0 (0.0)	0 (0.0)	0 (0.0)	0 (0.0)
H_2_FPEF Score (%)						
	Baseline	Follow-up	Baseline	Follow-up	Baseline	Follow-up
Low risk, *n* (%)	41 (89.1)	41 (89.1)	6 (75.0)	5 (62.5)	31 (88.6)	31 (88.6)
Intermediate risk, *n* (%)	2 (4.4)	3 (6.5)	2 (25.0)	3 (37.5)	4 (11.4)	4 (11.4)
High risk, *n* (%)	3 (6.5)	2 (4.4)	0 (0.0)	0 (0.0)	0 (0.0)	0 (0.0)
ABA Score						
	Baseline	Follow-up	Baseline	Follow-up	Baseline	Follow-up
Low risk, *n* (%)	36 (78.3)	35 (76.1)	4 (50.0)	4 (50.0)	28 (80.0)	27 (77.1)
Intermediate risk, *n* (%)	6 (13.0)	6 (13.0)	3 (37.5)	3 (37.5)	5 (14.3)	5 (14.3)
High risk, *n* (%)	4 (8.7)	5 (10.9)	1 (12.5)	1 (12.5)	2 (5.7)	2 (8.6)
HFA-PEFF Score (%)						
	Baseline	Follow-up	Baseline	Follow-up	Baseline	Follow-up
Low risk, *n* (%)	28 (60.9)	26 (56.5)	3 (37.5)	2 (25.0)	19 (54.3)	21 (60.0)
Intermediate risk, *n* (%)	17 (37.0)	19 (41.3)	4 (50.0)	6 (75.0)	15 (42.9)	12 (34.3)
High risk, *n* (%)	1 (2.2)	1 (2.2)	1 (12.5)	0 (0.0)	1 (2.9)	2 (5.7)

## Data Availability

The original contributions presented in this study are included in the article/Supplementary Materials. Further inquiries can be directed to the corresponding authors.

## Supplementary Materials

The following supporting information can be downloaded at: https://www.mdpi.com/article/10.3390/jcm15145739/s1, Table S1: Distribution of baseline HFA-PEFF risk categories according to natriuretic peptide availability; Table S2: Sensitivity Analysis: Heart Failure With Preserved Ejection Fraction Probability Scores Concordance Stratified by Natriuretic Peptide Availability; Table S3: Baseline Characteristics According to Natriuretic Peptide Availability; Table S4: Oncologic Treatments of the Longitudinal Subgroup; Table S5: Longitudinal Subgroup Characteristics at Baseline Stratified by Treatment Exposure; Table S6: Baseline and Follow-up Characteristics of the longitudinal subgroup according to treatment exposure; Table S7: Heart Failure With Preserved Ejection Fraction Probability Scores In The Longitudinal Subgroup.

## Abbreviations

The following abbreviations are used in this manuscript:

ACEi	Angiotensin-converting enzyme inhibitors
AF	Atrial fibrillation
ARB	Angiotensin receptor blockers
BB	Beta-blockers
BMI	Body Mass Index
BNP	B-type Natriuretic Peptide
CAD	Coronary artery disease
COPD	Chronic Obstructive Pulmonary Disease
eGFR	estimated Glomerular Filtration Rate
GDMT	Guideline-Directed Medical Therapy
HFpEF	Heart failure with preserved ejection fraction
HFrEF	Heart failure with reduced ejection fraction
IQR	Interquartile Range
IVS	Interventricular Septum
LAVI	Left atrial volume index
LVDTD	Left ventricular end-diastolic diameter
LVEF	Left Ventricular Ejection Fraction
LVMI	Left Ventricular Mass Index
MRA	Mineralocorticoid receptor antagonists
N	Number (of patients/sample size)
NT-proBNP	N-terminal pro-B-type Natriuretic Peptide
NYHA	New York Heart Association (Functional Classification)
RWT	Relative Wall Thickness
SD	Standard Deviation
SGLT2-i	Sodium-glucose cotransporter-2 inhibitor
sPAP	Systolic pulmonary artery pressure
