# Supplementary material for: Heart Failure with Preserved Ejection Fraction in Women with Breast Cancer Prior to Cancer Treatment: Insights from a Cardio-Oncology Assessment
Source: J Clin Med. 2026 Jul 22;15(14):5739. doi: 10.3390/jcm15145739 (PMC13413197; doi:10.3390/jcm15145739)
Supplement: Supplementary file 1 [file jcm-15-05739-s001.zip › jcm-4414408-supplementary.pdf]

Supplementary Materials:

Table S1. Distribution of Baseline HFA-PEFF Risk Categories According to Natriuretic Peptide Availability (n = 142).

Biomarker Availability	Low risk, *n* (%)	Intermediate risk, *n* (%)	High risk, *n* (%)	*p*-value*
No Biomarkers Available subgroup	54 (68.4%)	25 (31.6%)	0 (0.0%)	0.006
Biomarkers Available subgroup	29 (46.0%)	31 (49.2%)	3 (4.8%)	
Total	83 (58.5%)	56 (39.4%)	3 (2.1%)	

* *p*-value calculated using the Fisher-Freeman-Halton exact test for 2 x 3 contingency tables. Three patients were excluded from the baseline cohort because BMI data were unavailable.

Table S2. Sensitivity Analysis: Heart Failure With Preserved Ejection Fraction Probability Scores Concordance Stratified by Natriuretic Peptide Availability.

HFPEF Probability Score	No biomarkers data available (n = 82)	Biomarkers data available (n =63)
	Weighted κ (p-value)	Weighted κ (p-value)
H2FPEF (Points) vs. HFA-PEFF	0.102 (0.180)	0.252 (0.009)
H2FPEF (Points) vs. ABA Score	0.390 (<0.001)	0.614 (<0.001)
HFA-PEFF vs. ABA Score	0.090 (0.176)	0.152 (0.053)
H2FPEF (%) vs. HFA-PEFF	0.116 (0.091)	0.157 (0.028)
H2FPEF (%) vs. ABA Score	0.631 (<0.001)	0.592 (<0.001)

Table S3. Baseline Characteristics According to Natriuretic Peptide Availability*.

Variable	No biomarkers available subgroup (n = 82)	Biomarkers available subgroup (n =63)	p-value
	**Baseline**	**Baseline**	
Mean Age, years ± SD	57.1 ± 12.2	57.0 ± 12.0	0,960
Mean body mass index, kg/m^2 ± SD	25.2 ± 4.5	25.6 ± 5.3	0,722
Hemoglobin, g/dl ± SD	12.9 ± 1.6	12.3 ± 1.3	0,058
Atrial fibrillation history, *n* (%)	3 (4.0)	2 (3.0)	0,870
**NYHA Class, *n* (%)**			0,049
I, *n* (%)	80 (97.6)	55 (87.3)	
II, *n* (%)	2 (2.4)	7 (11.1)	
III, *n* (%)	0 (0.0)	1 (1.6)	
**HFA-ICOS Risk Assessment score**			-

Low risk, *n* (%)	0 (0.0)	35 (62.5)	
Medium risk, *n* (%)	0 (0.0)	18 (32.1)	
High risk, *n* (%)	0 (0.0)	3 (5.4)	
Very high risk, *n* (%)	0 (0.0)	0 (0.0)	
**Coexisting illness**			
Hypertension, *n* (%)	29 (35.4)	19 (30.2)	0,510
Dyslipidemia, *n* (%)	18 (22.0)	17 (27.0)	0,480
Diabetes, *n* (%)	5 (6.1)	8 (12.7)	0,170
COPD, *n* (%)	0 (0.0)	1 (1.6)	0,250
**Ongoing pharmacological treatment**			
ACEi/ARB, *n* (%)	17 (20.7)	12 (19.0)	0,800
BB, *n* (%)	13 (15.6)	11 (17.5)	0,667
Calcium antagonists, *n* (%)	0 (0.0)	0 (0.0)	
MRA, *n* (%)	0 (0.0)	0 (0.0)	
SGLT2-i, *n* (%)	1 (1.2)	0 (0.0)	
Statin therapy, *n* (%)	7 (8.5)	10 (15.9)	0,170
**Renal function**			
eGFR, ml/min/1.73 m^2 ± SD	92.2 ± 12.4	90.3 ± 14.7	0,530
**Echocardiographic data**			
LVEF, % ± SD	64.5 ± 4.3	64.4 ± 4.7	0,994
LVDTD mm ± SD	42.8 ± 4.2	44.5 ± 4.1	0,681
SIV, mm ± SD	9.4 ± 1.6	9.1 ± 1.5	0,370
RWT, *n* ± SD	0.4 ± 0.1	0.4 ± 0.1	0,410
LAVI, ml/m^2 ± SD	24.6 ± 8.1	22.5 ± 5.7	0,140
E/e', n ± SD	7.8 ± 2.0	7.7 ± 2.3	0,780
Septal e' < 7 cm/s or lateral e' < 10 cm/s, n (%)	24 (29.0)	27 (43.0)	0,089
sPAP, mmHg ± SD	26.8 ± 2.9	26.8 ± 4.1	0,890
LVMI, g/m^2 ± SD	68.6 ± 17.8	66.0 ± 14.6	0,340

*Absolute frequencies and percentages were calculated based on the total number of patients with available data for each specific variable

Table S4. Oncologic Treatments of the Longitudinal Subgroup.

Variable	Patients (N = 89)
Cumulative anthracycline dose, mg/m^2 ± SD	355.32 ± 32.3
Number of anti-HER2 cycles administered, *n* ± SD	17.5 ± 1.9
Left-sided radiotherapy, *n* (%)	39 (52,7)
Right-sided radiotherapy, *n* (%)	34 (46)
Whole breast/chest wall irradiation dose, Gy ± SD	41.0 ± 3.2
Boost irradiation dose, Gy ± SD	44.0 ± 9.5
**Endocrine therapy**	

Tamoxifen, *n* (%)	1 (2.6)
Aromatase inhibitor, *n* (%)	38 (97.4)

*Absolute frequencies and percentages were calculated based on the total number of patients with available data for each specific variable

Table S5. Longitudinal Subgroup Characteristics at Baseline Stratified by Treatment Exposure.

Variable	Anthracycline Group (*n* = 46)	Anti-HER2 Group (*n* = 8)	Combination Group (*n* =35)	p-value
	**Baseline**	**Baseline**	**Baseline**	
Mean Age, years ± SD	55.4 ± 11.6	61.1 ± 17.5	55.4 ± 10.2	0,420
Mean body mass index, kg/m² ± SD	25.2 ± 4.5	26.6 ± 7.5	25.1 ± 4.9	0,722
Hemoglobin, g/dl ± SD	12.3 ± 1.3	13.7 ± 1.5	12.3 ± 1.4	0,027
Atrial fibrillation history, *n* (%)	2 (4.4)	0 (0.0)	1 (2.9)	1.000
**NYHA Class, *n* (%)**				0,057
I, *n* (%)	40 (87.0)	8 (100.0)	35 (100.0)	
II, *n* (%)	6 (13.0)	0 (0.0)	0 (0.0)	
III, *n* (%)	0 (0.0)	0 (0.0)	0 (0.0)	
**Coexisting illness**				
Hypertension, *n* (%)	11 (23.9)	2 (25.0)	10 (28.6)	0,934
Dyslipidemia, *n* (%)	11 (23.9)	1 (12.5)	8 (22.9)	0,932
Diabetes, *n* (%)	2 (4.4)	1 (12.5)	2 (5.7)	0,482
COPD, *n* (%)	0 (0.0)	0 (0.0)	1 (2.9)	0,483
**Ongoing pharmacological treatment**				
ACEi/ARB, *n* (%)	7 (15.2)	1 (12.5)	7 (20.0)	0,911
BB, *n* (%)	6 (13.0)	0 (0)	7 (20.0)	0,667
Calcium antagonists, *n* (%)	0 (0.0)	0 (0.0)	0 (0.0)	
MRA, *n* (%)	0 (0.0)	0 (0.0)	0 (0.0)	
SGLT2-i, *n* (%)	0 (0.0)	0 (0.0)	0 (0.0)	
Statin therapy, *n* (%)	6 (13.0)	1 (12.5)	3 (8.6)	0,887
**Renal function**				
eGFR, ml/min/1.73 m² ± SD	91.2 ± 17.1	93.9 ± 9.9	92.0 ± 14.8	0,910
**Biomarkers**				
Median NT-proBNP, pg/mL (IQR)	116.0 (62.0-325.0)	29.0 (10.0-48.0)	122.0 (75.0-207.0)	0,189
Median BNP, pg/mL (IQR)	29.0 (13.0-68.0)	50.0 (10.0-111.0)	24.0 (12.0-34.5)	0,590
**Echocardiographic data**				
LVEF, % ± SD	64.5 ± 5.4	64.5 ± 4.3	64.4 ± 4.7	0,994
LVDTD mm ± SD	44.5 ± 4.6	42.8 ± 4.2	44.5 ± 4.1	0,681
SIV, mm ± SD	9.3 ± 1.5	9.6 ± 1.8	9.1 ± 1.4	0,555
RWT, *n* ± SD	0.4 ± 0.1	0.4 ± 0.1	0.4 ± 0.1	0,172
LAVI, ml/m² ± SD	21.9 ± 5.3	21.9 ± 6.0	24.7 ± 9.1	0,307

E/e′, *n* ± SD	7.5 ± 2.4	8.4 ± 2.4	7.9 ± 1.7	0,497
Septal e′ < 7 cm/s or lateral e′ < 10 cm/s, *n* (%)	16 (34.8)	5 (62.5)	13 (37.1)	0,413
sPAP, mmHg ± SD	27.3 ± 3.4	24.9 ± 3.0	26.4 ± 3.1	0,128
LVMI, g/m^2 ± SD	65.2 ± 13.2	69.7 ± 13.6	66.2 ± 12.4	0,656

Table S6. Baseline and Follow-up Characteristics of the longitudinal subgroup according to treatment exposure.

**Variable**	**Anthracycline Group (*n* = 46)**		**p-value**	**Anti-HER2 Group (*n* = 8)**		**p-value**	**Combination Group (*n* =35)**		**p-value**
	**Base line**	**FU**		**Base line**	**FU**		**Base line**	**FU**	
Mean Age, years ± SD	55.4 ± 11.6	56.4 ± 11.6	0,000	61.1 ± 17.5	62.1 ± 17.5	0,000	55.4 ± 10.2	56.5 ± 10.2	0,000
Mean body mass index, kg/m^2 ± SD	25.2 ± 4.5	25.7 ± 4.7	0,101	26.6 ± 7.5	25.7 ± 6.8	0,105	25.1 ± 4.9	25.3 ± 5.4	0,645
Hemoglobin, g/dl ± SD	12.3 ± 1.3	12.4 ± 1.2	0,205	13.7 ± 1.5	13.6 ± 2.1	0,624	12.3 ± 1.4	12.6 ± 1.0	0,918
Atrial fibrillation history, *n* (%)	2 (4.4)	2 (4.4)	-	0 (0.0)	0 (0.0)	-	1 (2.9)	1 (2.9)	-
**NYHA Class, *n* (%)**			0,157			0,317			0,317
I, *n* (%)	40 (87.0)	42 (91.3)		8 (100.0)	7 (87.5)		35 (100.0)	34 (97.1)	
II, *n* (%)	6 (13.0)	4 (8.7)		0 (0.0)	1 (12.5)		0 (0.0)	1 (2.9)	
III, *n* (%)	0 (0.0)	0 (0.0)		0 (0.0)	0 (0.0)		0 (0.0)	0 (0.0)	
**Coexisting illness**									
Hypertension, *n* (%)	11 (23.9)	11 (23.9)	-	2 (25.0)	2 (25.0)	-	10 (28.6)	10 (28.6)	-
Dyslipidemia, *n* (%)	11 (23.9)	11 (23.9)	-	1 (12.5)	1 (12.5)	-	8 (22.9)	8 (22.9)	-
Diabetes, *n* (%)	2 (4.4)	2 (4.4)	-	1 (12.5)	1 (12.5)	-	2 (5.7)	2 (5.7)	-
COPD, *n* (%)	0 (0.0)	0 (0.0)	-	0 (0.0)	0 (0.0)	-	1 (2.9)	1 (2.9)	-
**Ongoing pharmacological treatment**									
ACEi/ARB, *n* (%)	7 (15.2)	9 (19.6)	0,157	1 (12.5)	2 (25.0)	0,317	7 (20.0)	8 (22.9)	0,564
BB, *n* (%)	6 (13.0)	15 (32.6)	0,029	0 (0)	2 (25.0)	0,157	7 (20.0)	9 (25.7)	0,514
Calcium antagonists, *n* (%)	0 (0.0)	0 (0.0)	-	0 (0.0)	0 (0.0)	-	0 (0.0)	0 (0.0)	-
MRA, *n* (%)	0 (0.0)	0 (0.0)	-	0 (0.0)	1 (12.5)	0,317	0 (0.0)	0 (0.0)	-
SGLT2-i, *n* (%)	0 (0.0)	0 (0.0)	-	0 (0.0)	0 (0)	-	0 (0.0)	0 (0.0)	-
Statin therapy, *n* (%)	6 (13.0)	11 (23.9)	0,025	1 (12.5)	3 (37.5)	0,157	3 (8.6)	8 (22.9)	0,025

**Renal function**									
eGFR, ml/min/1.73 m² ± SD	91.2 ± 17.1	89.8 ± 17.3	0,804	93.9 ± 9.9	93.4 ± 10.8	0,063	92.0 ± 14.8	90.7 ± 16.2	0,276
**Biomarkers**									
Median NT-proBNP, pg/mL (IQR)	116.0 (62.0-325.0)	113.5 (58.0-179.0)	0,156	29.0 (10.0-48.0)	111.0 (111.0-111.0)	0,317	122.0 (75.0-207.0)	41.5 (22.0-144.0)	0,317
Median BNP, pg/mL (IQR)	29.0 (13.0-68.0)	35.0 (30.0-55.0)	0,265	50.0 (10.0-111.0)	10.0 (7.5-16.0)	0,317	24.0 (12.0-34.5)	19.0 (11.0-43.5)	0,894
**Echocardiographic data**									
LVEF, % ± SD	64.5 ± 5.4	64.9 ± 4.4	0,595	64.5 ± 4.3	63.8 ± 4.4	0,688	64.4 ± 4.7	64.3 ± 4.3	0,959
LVDTD	44.5 ± 4.6	44.7 ± 4.3	0,828	42.8 ± 4.2	44.4 ± 3.6	0,377	44.5 ± 4.1	43.8 ± 4.3	0,216
SIV, mm ± SD	9.3 ± 1.5	9.2 ± 1.4	0,754	9.6 ± 1.8	8.9 ± 1.0	0,265	9.1 ± 1.4	8.9 ± 1.2	0,173
RWT, *n* ± SD	0.4 ± 0.1	0.4 ± 0.1	0,958	0.4 ± 0.1	0.4 ± 0.0	0,261	0.4 ± 0.1	0.4 ± 0.1	0,609
LAVI, ml/m² ± SD	21.9 ± 5.3	20.7 ± 7.6	0,940	21.9 ± 6.0	20.5 ± 6.3	0,807	24.7 ± 9.1	23.4 ± 7.0	0,783
E/e’, n ± SD	7.5 ± 2.4	7.2 ± 1.9	0,374	8.4 ± 2.4	8.2 ± 2.1	0,871	7.9 ± 1.7	8.0 ± 1.9	0,706
Septal e’ < 7 cm/s or lateral e’ < 10 cm/s, *n* (%)	16 (34.8)	17 (36.7)	0,763	5 (62.5)	5 (62.5)	1,000	13 (37.1)	12 (34.3)	0,706
sPAP, mmHg ± SD	27.3 ± 3.4	27.0 ± 2.4	0,628	24.9 ± 3.0	28.1 ± 7.2	0,289	26.4 ± 3.1	26.3 ± 3.3	0,716
LVMI, g/m² ± SD	65.2 ± 13.2	65.5 ± 14.0	0,892	69.7 ± 13.6	66.1 ± 9.5	0,429	66.2 ± 12.4	63.5 ± 12.1	0,241

Table S7. Heart Failure With Preserved Ejection Fraction Probability Scores In The Longitudinal Subgroup.

	**H_2FPEF Score (points)**			**H_2FPEF Score (%)**			**ABA Score (%)**			**HFA-PEFF Score**		
	**Base line**	**FU**	**p-value**	**Base line**	**FU**	**p-value**	**Base line**	**FU**	**p-value**	**Base line**	**FU**	**p-value**
Low risk, *n* (%)	59 (66.3)	57 (64.0)	0.82	78 (87.6)	77 (86.5)	0.51	68 (76.4)	66 (74.2)	0.14	50 (56.2)	49 (55.1)	0.98
Intermediate risk, *n* (%)	29 (32.6)	31 (34.8)		8 (9.0)	10 (11.2)		14 (15.7)	14 (15.7)		36 (40.5)	37 (41.6)	
High risk, *n* (%)	1 (1.1)	1 (1.1)		3 (3.4)	2 (2.3)		7 (7.9)	9 (10.1)		3 (3.4)	3 (3.4)	
